# Decrease of Cardiac Parkin Protein in Obese Mice

**DOI:** 10.3389/fcvm.2019.00191

**Published:** 2020-01-20

**Authors:** Amandine Thomas, Stefanie Marek-Iannucci, Kyle C. Tucker, Allen M. Andres, Roberta A. Gottlieb

**Affiliations:** ^1^Cedars-Sinai Medical Center, Smidt Heart Institute, Los Angeles, CA, United States; ^2^Department of Molecular and Cell Biology, University of California, Berkeley, Berkeley, CA, United States

**Keywords:** mitophagy, Parkin, obesity, ischemia/reperfusion, myocardium, mitochondria

## Abstract

Mitophagy plays a major role in heart physiology. Impairment of Parkin-dependent mitophagy in heart is known to be deleterious. Obesity is a known cardiovascular risk factor. Impaired autophagy has been reported in models of obesity or hyperlipidemia/hypercholesterolemia; however less is known regarding obesity and mitophagy. The aim of this study was to evaluate the regulation of Parkin expression in hearts of mice fed a high fat diet. Interestingly, we found a significant decrease in Parkin protein in hearts of HFD mice compared those fed a low-fat diet. This was associated with mitochondrial dysfunction in the context of ischemia/reperfusion (I/R). This downregulation was not associated with a decrease in Parkin mRNA expression. We did not detect any change in the degradation rate of Parkin and only a slight decrease in its translation. The reduction of Parkin protein abundance in HFD hearts remains a mystery and will need further studies. However, Parkin depletion in the setting of obesity may contribute to cardiovascular risk.

## Introduction

Mitochondrial clearance through mitophagy is a major element of mitochondrial homeostasis and plays an important role in maintaining cardiac well-being at baseline as well as during stress (1). Mitophagy occurs through different pathways involving Parkin, BNIP3, or FUNDC1. These appear to be complementary and differentially activated according to the stimulus (2, 3). Parkin-mediated mitophagy is generally triggered by mitochondrial inner membrane depolarization, which leads to PINK1 accumulation on the outer membrane and phosphorylation of targets that recruit Parkin. Parkin-dependent mitophagy has been well studied in the context of myocardial injury after ischemia/reperfusion (I/R) (4). Its role in the heart has been reevaluated in the light of the fact that Parkin deficiency at baseline did not induce cardiac dysfunction; however, Parkin is required for cardioprotection by ischemic preconditioning or statin administration (5, 6) and we previously reported that diet-induced obesity increases ischemic injury (7). Moreover, Parkin deficiency increases severity of ischemia/reperfusion (I/R) injury (8). Interestingly, Parkin plays an important role in the heart's transition from fetal to postnatal life involving a metabolic switch from carbohydrates to fatty acids and amino acids for fuel utilization; this highlights its potential significance in metabolic remodeling of mitochondria (4). Related to that, obesity is known to induce metabolic reprogramming of mitochondria as well as mitochondrial dysfunction (3). However, little is known about the regulation of cardiac mitophagy in the context of obesity. The aim of this study was to examine how Parkin-mediated mitophagy was regulated in a model of diet-induced obesity in mice.

## Methods

### Animals and Experimental Design

Eight-week-old male C57Bl/6J mice were housed under standard conditions in conventional cages with *ad libitum* food and water. Ambient temperature was maintained at 20–22°C. The mice were fed a low-fat diet (LFD: 10% energy derived from fat; D12450b; Research Diets) or a high-fat diet (HFD: 60% energy derived from fat; D12492; Research Diets) for 12 weeks. For the inhibition of proteasome and autophagy, HFD mice were treated, respectively, with intraperitoneal injection of Bortezomib (1 mg/kg) and Chloroquine (50 mg/kg). Mice were sacrificed 6 h after injections.

### Isolated Heart Perfusion

Hearts from anesthetized mice (i.p. pentobarbital 70 mg/kg) were rapidly excised and cannulated onto the Langendorff apparatus and perfused in a retrograde manner with Krebs-Henseleit bicarbonate buffer consisting of: (in g/L) NaCl 6.9, KCl 0.35, MgSO_4_ 0.14, KH_2_PO_4_ 0.16, NaHCO_3_ 2.1, CaCl_2_ 0.37, glucose 2.0, gassed with 95%O_2_ /5%CO_2_ (pH 7.4). The buffer reservoir height was adjusted to achieve a perfusion pressure of 60–80 mm Hg and perfusate temperature was maintained at 37°C. Hearts were allowed to stabilize for 15 min prior to induction of global no-flow ischemia via cessation of perfusion for 30 min. Temperature was maintained during ischemia by immersing the heart in perfusate maintained at 37°C. Hearts were then reperfused by restoring flow and maintained for 30 min. Pre-ischemic and reperfusion flow rates were measured. At the end of the experiment atria and ventricles were rapidly excised and immediately snap frozen in liquid nitrogen or further processed for mitochondrial isolation. For infarct size measurement, the hearts were cut into five transverse slices. Each slice was incubated for 20 min in 1% triphenyltetrazolium chloride solution at 37°C to differentiate infarcted from viable myocardial areas. Extension of the area of necrosis was quantified by planimetric analysis (ImageJ software).

### Western Blot Analysis

Total cell lysates were obtained after lysing frozen heart samples (~50 mg) in ice-cold RIPA buffer containing: (in mM) Tris-HCl 50, NaCl 150, EDTA 2, NaF 50, and detergents Na-deoxycholate 0.5%, SDS 0.1%, NP40 1%, and protease inhibitors cocktail (Complete, Roche). Mitochondrial fractions were obtained after homogenization of fresh heart samples (30–50 mg) in ice-cold mitochondrial isolation buffer (250 mM sucrose; 1 mM EDTA; 10 mM HEPES, pH 7.4) containing protease and phosphatase inhibitors (Complete, Roche). Nuclei and unbroken cells were eliminated by low-speed spin (1,000 g, 4°C, 10 min). Postnuclear supernatant was centrifuged (7,000 g, 4°C, 15 min) to obtain the final mitochondria-enriched pellet and supernatant (crude cytosol). The mitochondria-enriched fraction was resuspended in isolation buffer and centrifuged (7,000 g, 4°C, 5 min). The final pellet was resuspended in ice cold RIPA buffer with inhibitors. Both total cell lysate and mitochondrial fractions were probed with primary antibodies against Parkin (sc-32282, Santa Cruz Biotechnology), Ubiquitinated protein (ab-7780, Abcam), HSP60 (Cell signaling #12165) and CHOP (Cell signaling #5554). Bands were visualized by enhanced chemiluminescence and quantified using Image lab (Biorad). All protein expression levels have been normalized to ponceau staining.

### Polysome Profiling

Polysome profiling has been done as previously described (9). Briefly, heart samples were homogenized in a buffer containing: (in mM) KCl 100, Tris 20, MgCl_2_ 5, pH 7.5, with 0.4% NP-40, 100 μg/ml cycloheximide and 0.1 U/μl RNase inhibitor (Invitrogen). Homogenates were incubated 15 min on ice and centrifuged at 14,000 rpm for 15 min at 4°C. The supernatants were loaded onto 15–50% (w/v) sucrose gradients and centrifuged at 37,000 rpm in a Beckman SW41 Ti rotor for 2 h at 4°C. Gradient fractions were collected with a BioLogic LP System. Total RNA was isolated from fractions with Trizol following the manufacturer's suggested procedure.

### RNA Purification and qRT-PCR

RNA was extracted from snap-frozen heart (~25 mg) using Trizol RNA isolation reagent. Total RNA (0.5 μg) was reverse-transcribed and quantitative real-time PCR was then performed with SYBR Green Core Kit on a thermal cycler (Bio-Rad). mRNA expression was normalized to 18S or Rplp0 mRNA content and expressed as fold change compared to control mice using the ΔΔ CT method. Primer sequences are shown in Table 1.

**Table 1:** Primer Sequences.

	**Forward**	**Reverse**
Parkin	CGTGTGTAGCTGGCTGTCCCAA	ACCTCCCATTTGCAGCACGCA
HSP60	CCCGCAGAAATGCTTCGACT	ACTTTGCAACAGTGACCCCA
mt-HSP70	TGCCTCCAATGGTGATGCTT	CAGCATCCTTAGTGGCCTGT
18S	GACTCAACACGGGAAACCTC	AGACAAATCGCTCCACCAAC
Rplp0	TCTGGAGGGTGTCCGCAACG	GCCAGGACGCGCTTGTACCC

### Statistical Analysis

All data are expressed as mean ± SEM. Statistical analysis was performed using Graphpad Prism 6 software package for Windows with two-tailed unpaired Student's test (LFD vs. HFD) or two-way ANOVA with multiple comparisons followed by *post hoc* Fisher's LSD test (LFD vs. HFD on either basal or I/R conditions). Differences between groups were considered statistically significant when *p* < 0.05.

## Results

**Figure 1:**
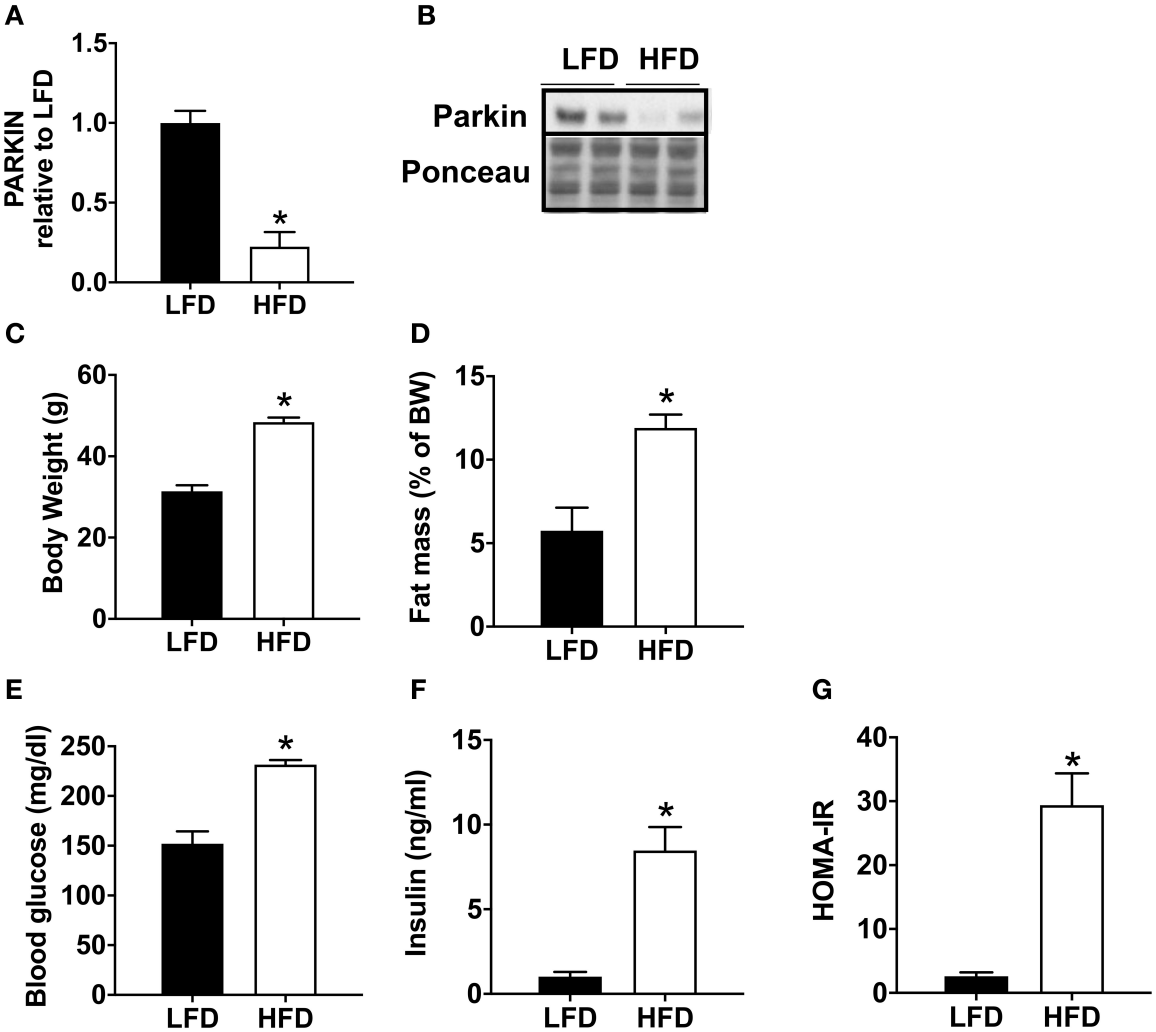
Decrease of cardiac Parkin protein level in mice after 12 weeks of HFD. The protein expression of Parkin was quantified by densitometric analysis **(A)** after Western blot analysis **(B)** in LFD and HFD mice at baseline (no ischemia reperfusion, fed *ad libitum*). Body weight **(C)** was monitored after 12 weeks of LFD or HFD. The fat mass was calculated after measurement of adipose tissue mass after sacrifice **(D)**. After 1 weeks of HFD, plasma glucose **(E)** and insulin **(F)** levels were determined in mice fasted for 6 h and HOMA-IR was calculated **(G)**. Results (*n* = 5–8/group) are expressed in mean ± SEM; *p < 0.05 vs. LFD.

Mice fed with a high-fat diet (HFD) exhibit a significant decrease in Parkin protein level (Figures 1A,B). In order to validate the model of diet-induced obesity, metabolic phenotype parameters were evaluated. The HFD fed mice presented a higher body weight (Figure 1C) and increased fat mass (Figure 1D). Blood glucose (Figure 1E) and insulin levels were higher (Figure 1F), leading to an increase in HOMA-IR (Figure 1G).

**Figure 2:**
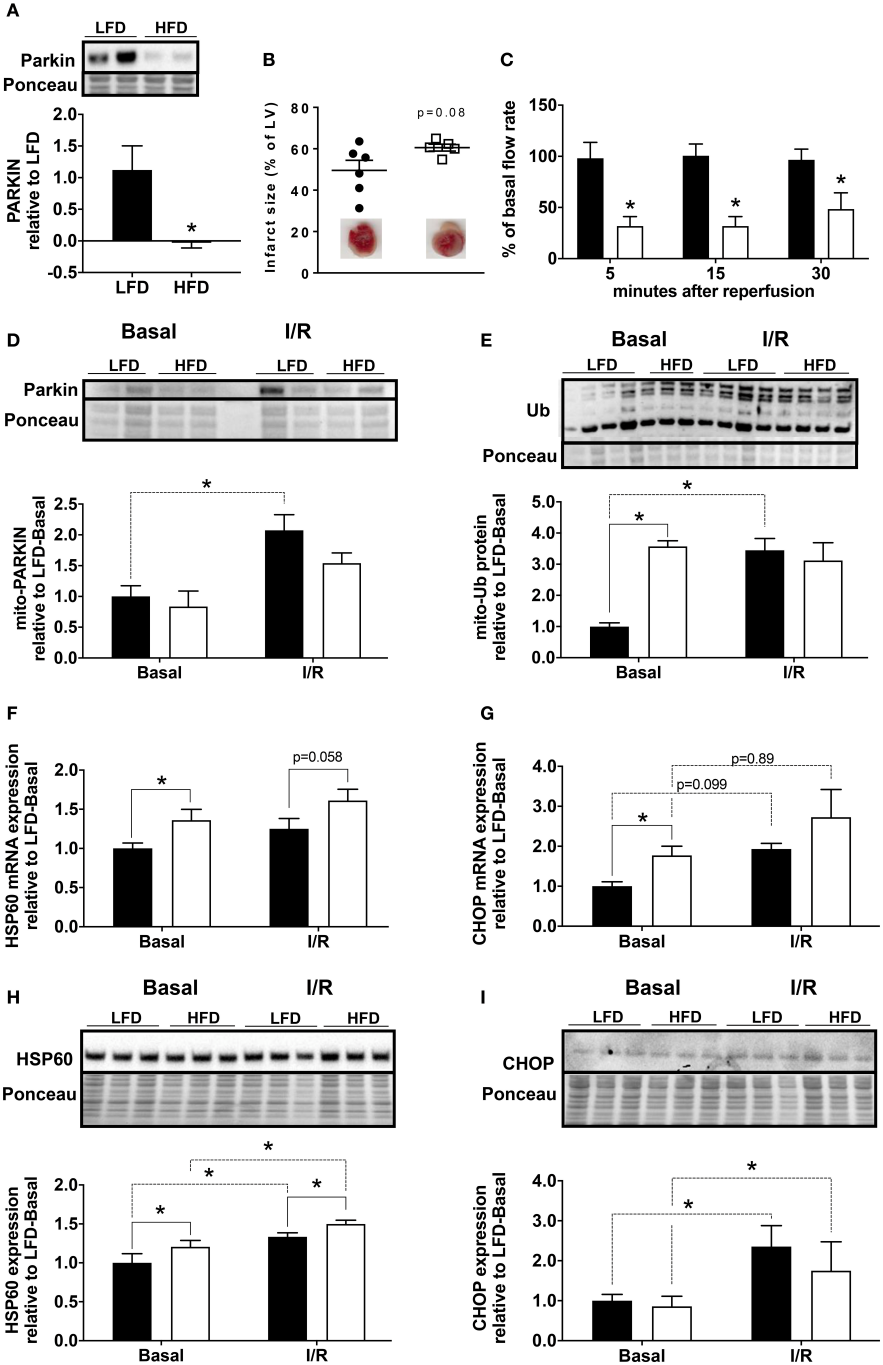
Loss of Parkin, cardiac and mitochondrial homeostasis alteration in HFD mice subjected to ischemia/reperfusion (I/R). Protein expression of Parkin was quantified by densitometric analysis of Western blots of heart lysates **(A)** from LFD and HFD mice after I/R. Infarct size was determined by colorimetry and quantified by planimetry **(B)**, examples of heart slices are shown on the graph. Flow rate recovery was measured at indicated time point after reperfusion **(C)**. Parkin **(D)** and ubiquitinated proteins **(E)** were detected by Western blot in mitochondrial extracts from hearts of LFD and HFD mice after I/R. Cardiac expression of genes involved in mitochondrial stress: HSP60 **(F)** and CHOP **(G)** were measured by RT-qPCR. The HSP60 **(H)** and CHOP **(I)** protein expression levels were quantified by densitometric analysis of Western blots. Results (*n* = 4–6/group) are expressed in mean ± SEM; *p < 0.05.

To determine if Parkin level changed acutely during cardiac ischemia and reperfusion, we isolated hearts from low fat diet (LFD) and HFD mice and subjected them to 30 min global ischemia and 3 h reperfusion via Langendorff perfusion. We found that the level of Parkin protein remained low in the hearts of HFD mice compared to LFD after I/R (Figure 2A). In our acute I/R model, we saw a modest trend toward increased infarct size (Figure 2B) and a significant decrease of coronary reflow in hearts of HFD mice (Figure 2C). Pre-ischemic coronary flows were not different between LFD and HFD mice (data not shown). Under basal conditions, the level of mitochondria-associated Parkin is low in hearts of both LFD and HFD mice; however, after I/R, Parkin translocated to mitochondria only in the LFD mice (Figure 2D). Consistent with this, the quantity of ubiquitinated protein in the mitochondrial fraction increased after I/R only in the LFD group (Figure 2E). Interestingly, mitochondrial protein ubiquitin was already high in the basal state in HFD mice. This likely reflects reduced clearance of Ub-tagged mitochondrial proteins via mitophagy or proteasomal degradation. As mitochondrial dysfunction can trigger the mitochondrial unfolded protein response (10), we measured mRNA and protein level for HSP60 and CHOP. mRNA levels of both HSP60 (Figure 2F) and CHOP (Figure 2G) are increased in the HFD group under basal conditions with a more pronounced change for CHOP mRNA. I/R tends to upregulate both targets, but no significant differences between LFD and HFD are observed. The densitometry analysis showed that the increase of HSP60 is maintained at the protein level (Figure 2H) with a slight but statistically significant upregulation of the protein upon HFD and I/R. The increase of CHOP mRNA level is not reflected by an increase of its protein level (Figure 2I) under basal conditions. Like CHOP mRNA, CHOP protein level is upregulated by I/R but no significant differences appear between LFD and HFD groups.

**Figure 3:**
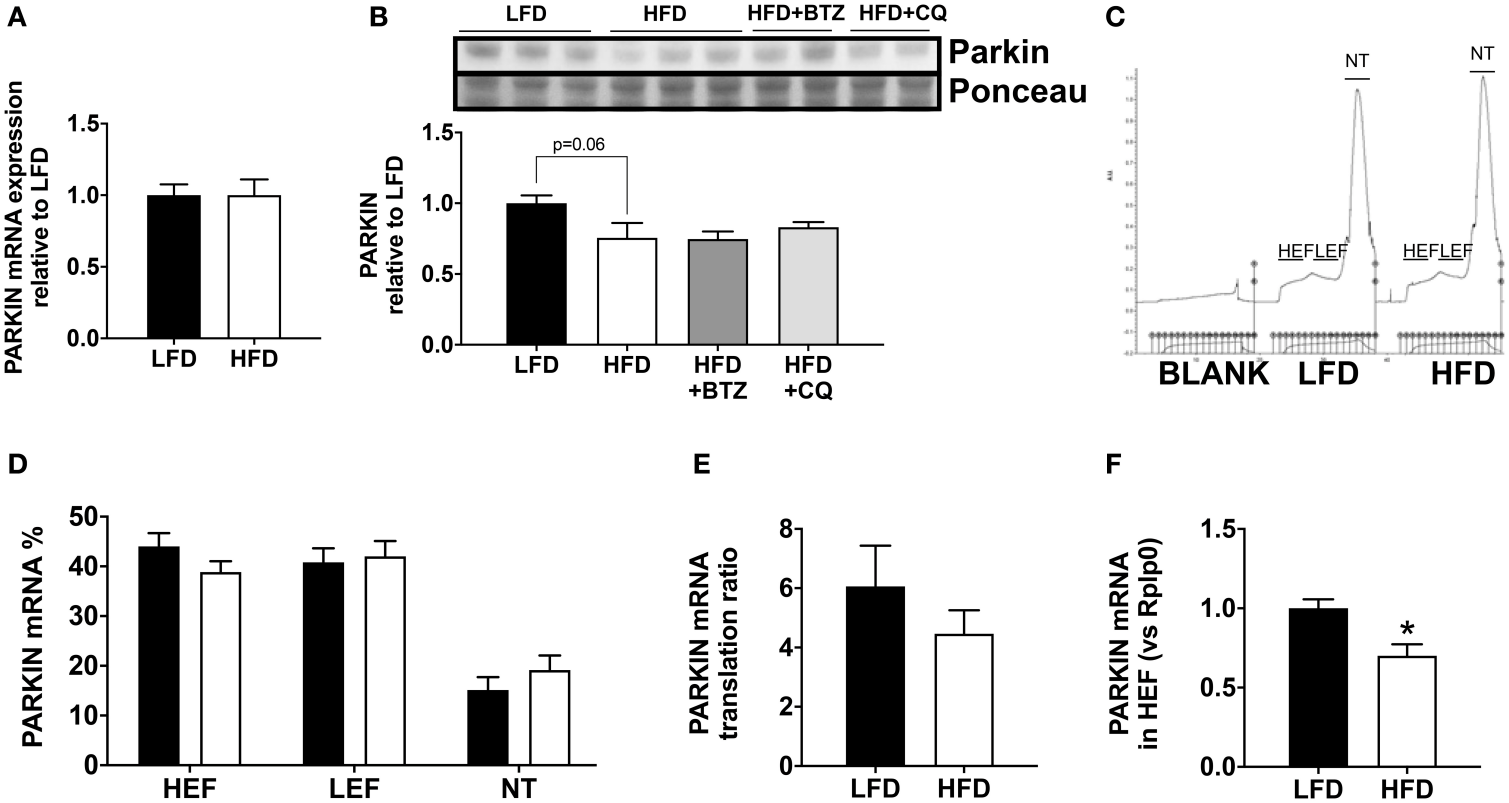
Regulation of Parkin protein abundance. The cardiac mRNA expression of Parkin **(A)**. The protein expression of Parkin was analyzed Western blot analysis **(B)** in LFD and HFD mice and HFD mice treated with Bortezomib (1 mg/kg) or Chloroquine (50 mg/kg). UV densitometry tracing of RNA in the sucrose gradient for polysome profiling **(C)**. Polysome profiling to detect distribution of Parkin mRNA in high-efficiency (HEF) and low-efficiency (LEF) polysomes and the non-translating (NT) fraction **(D)**. Quantitation of Parkin mRNA translation ratio [(HEF + LEF)/NT] **(E)**. Quantitation of Parkin mRNA level in the HEF **(F)**. Results (*n* = 5–6/group) are expressed in mean ± SEM.

In order to understand the basis for reduced Parkin protein in HFD mice, we assessed the mRNA expression of Parkin and found no difference between the groups (Figure 3A). The observed lack of change in mRNA expression in our model suggested increased Parkin degradation. To determine if the decrease in Parkin was related to increased protein degradation, we treated mice for 6 h with either bortezomib or chloroquine *in vivo* to block, respectively, proteasome activity or autophagic flux. Neither treatment restored Parkin protein levels in HFD mice hearts (Figure 3B). We then analyzed if there was a change in translational activity for Parkin, using polysome profiling (9). When we consider the mRNA distribution, we observed that Parkin mRNA is less abundant in the translating fraction and more present in the non-translating fraction (Figures 3C–E). This is confirmed by the significant decrease of Parkin mRNA in the high efficiency translating fraction (HEF) (Figure 3F).

## Discussion

Few studies have examined the regulation of Parkin protein in the setting of obesity. Parkin is upregulated in vascular walls (11) or adipose tissue (12) but decreased in the brain substantia nigra (SN) (13) of obese or diabetic mice. In liver, studies show both an increase (14) or a decrease (15) in Parkin level upon obesity. Contrary to our results, Tong et al. observed an increase in cardiac parkin protein during HFD consumption, although their paper did not indicate how many weeks of HFD were performed prior the analysis of Parkin (16). In their study, Parkin KO mice developed more severe cardiac hypertrophy and cardiac diastolic dysfunction in response to HFD feeding, suggesting that upregulation of Parkin-dependent mitophagy is a homeostatic response to HFD. These data suggest that obesity affects expression of Parkin protein and mitophagy capacity. Interestingly, these changes appear to be tissue specific and affected by the duration of the HFD. Further studies are needed to understand the effect of Parkin expression variations. In our case, we demonstrated a significant decrease of Parkin level in hearts of obese mice fed HFD for 12 weeks. The loss of Parkin is known to be deleterious for heart physiology (8, 17). Under basal conditions, Parkin deficient mice did not present a major phenotype. However, ischemic preconditioning cannot protect Parkin-deficient mice from ischemia/reperfusion injury (6). Also, these mice develop more severe cardiac remodeling after permanent ligation of left ventricular artery (8). Overall, the lack of Parkin protein in hearts of obese mice is associated with myocardial injury after I/R as reflected by the trend toward increased infarct size and the no-reflow phenomenon in the HFD group. The decrease of total Parkin level may be responsible for the impairment of its translocation to the mitochondria in the context of an ischemic stress, as we observed less Parkin translocated to the mitochondria upon I/R. Moreover, the latter seems to be associated with an increase in mitochondrial stress marker in a basal state. This is in agreement with the idea that Parkin plays a major role in mitochondrial stress, with or without apparent cardiac dysfunction (4). We cannot exclude compensation by other mitophagy pathways that may mitigate injury linked to the reduction of Parkin in the hearts of HFD mice. This result is consistent with the results of Khang et al. (13), who described a decrease in Parkin protein level in the substantia nigra of HFD or db/db mice without any change in mRNA expression of Parkin. Interestingly, they showed that insulin treatment in SH-SY5Y cell line induced a decrease of Parkin, suggesting a role for insulin signaling in the regulation of Parkin protein expression. We hypothesize that this modest decrease in translational efficiency of Parkin mRNA can result in a gradual decrease in Parkin protein, as well as a limited ability to rapidly upregulate Parkin translation in response to stress in HFD mice. However, further studies are needed to understand how Parkin is regulated in the context of obesity.

## Conclusion

In conclusion, this paper showed a substantial reduction of Parkin protein level in the hearts of HFD mice, although we were unable to discern the mechanism. Moreover, while Parkin is known to initiate mitophagy (and perhaps other unrecognized targets) via ubiquitination, little is known regarding regulation of Parkin abundance itself.

## Data Availability Statement

All datasets generated for this study are included in the article/supplementary material.

## Ethics Statement

Animal experiments were performed in accordance with the Institutional Animal Care and Use Committee of Cedars-Sinai Medical Center (IACUC5000).

## Author Contributions

AT performed experiments, analyzed data, and wrote the manuscript. SM-I performed experiments, analyzed data, and critically reviewed the manuscript. KT performed experiments and contributed to discussion. AA performed experiments and contributed to discussion. RG supervised the project and edited the manuscript.

### Conflict of Interest

The authors declare that the research was conducted in the absence of any commercial or financial relationships that could be construed as a potential conflict of interest.
